# *De novo* assembly and characterization of bark transcriptome using Illumina sequencing and development of EST-SSR markers in rubber tree (*Hevea brasiliensis* Muell. Arg.)

**DOI:** 10.1186/1471-2164-13-192

**Published:** 2012-05-18

**Authors:** Dejun Li, Zhi Deng, Bi Qin, Xianghong Liu, Zhonghua Men

**Affiliations:** 1 Key Laboratory of Biology and Genetic Resources of Rubber Tree, Ministry of Agriculture, Rubber Research Institute, Chinese Academy of Tropical Agricultural Sciences, Danzhou, Hainan, 571737, China; 2 Baotou Teachers College, Science Road, Qingshan district, Baotou, Inner Mongolia, 014030, China

**Keywords:** Rubber tree, Transcriptome, EST-SSR, Illumina paired-end sequencing, *de novo* assembly, Bark

## Abstract

**Background:**

In rubber tree, bark is one of important agricultural and biological organs. However, the molecular mechanism involved in the bark formation and development in rubber tree remains largely unknown, which is at least partially due to lack of bark transcriptomic and genomic information. Therefore, it is necessary to carried out high-throughput transcriptome sequencing of rubber tree bark to generate enormous transcript sequences for the functional characterization and molecular marker development.

**Results:**

In this study, more than 30 million sequencing reads were generated using Illumina paired-end sequencing technology. In total, 22,756 unigenes with an average length of 485 bp were obtained with *de novo* assembly. The similarity search indicated that 16,520 and 12,558 unigenes showed significant similarities to known proteins from NCBI non-redundant and Swissprot protein databases, respectively. Among these annotated unigenes, 6,867 and 5,559 unigenes were separately assigned to Gene Ontology (GO) and Clusters of Orthologous Group (COG). When 22,756 unigenes searched against the Kyoto Encyclopedia of Genes and Genomes Pathway (KEGG) database, 12,097 unigenes were assigned to 5 main categories including 123 KEGG pathways. Among the main KEGG categories, metabolism was the biggest category (9,043, 74.75%), suggesting the active metabolic processes in rubber tree bark. In addition, a total of 39,257 EST-SSRs were identified from 22,756 unigenes, and the characterizations of EST-SSRs were further analyzed in rubber tree. 110 potential marker sites were randomly selected to validate the assembly quality and develop EST-SSR markers. Among 13 *Hevea* germplasms, PCR success rate and polymorphism rate of 110 markers were separately 96.36% and 55.45% in this study.

**Conclusion:**

By assembling and analyzing *de novo* transcriptome sequencing data, we reported the comprehensive functional characterization of rubber tree bark. This research generated a substantial fraction of rubber tree transcriptome sequences, which were very useful resources for gene annotation and discovery, molecular markers development, genome assembly and annotation, and microarrays development in rubber tree. The EST-SSR markers identified and developed in this study will facilitate marker-assisted selection breeding in rubber tree. Moreover, this study also supported that transcriptome analysis based on Illumina paired-end sequencing is a powerful tool for transcriptome characterization and molecular marker development in non-model species, especially those with large and complex genomes.

## Background

Natural rubber is one of the most important raw materials for many industries, and it cannot be replaced by synthetic alternatives due to its unique properties, such as resilience, elasticity, impact and abrasion resistance, efficient heat dispersion and malleability at cold temperature [1,2]. Among over 2,000 plant species recognized for producing rubber, *Hevea brasiliensis* Muell. Arg. is the only species cultivated commercially for natural rubber. *H. brasiliensis* is a cross-pollinated, diploid (2n = 2× = 36) and perennial plant with a large genome size (~2100 Mb) [3]. Despite growing demand and high-yield potential, the production of natural rubber is relatively low, especially in China. Biotic and abiotic stresses, such as tapping panel dryness (TPD), powdery mildew, leaf blight, low temperature, strong wind and drought, are major yield-limiting factors on natural rubber production. The combination of conventional and modern breeding technologies will be helpful to increase the yield of rubber tree [4]. However, very limited genomic resources are available for rubber tree, which restricted the development of modern breeding technologies.

Various genomic tools have facilitated the development of improved genotypes/varieties in several crop species [5,6]. In rubber tree, expressed sequence tags (ESTs) and molecular markers have been developed, but the functional genomic studies are still in their infancy. Currently, there are only 37,745 rubber tree ESTs available in the national center for biotechnology information (NCBI) database (as of Dec 2011). Most of these ESTs were generated with the aim to identify the candidate genes involved in various abiotic and biotic stress responses and rubber biosynthesis [7-20], whereas only superoxide dismutase was further studied by using transgenic approaches [21-23]. Among these 37,745 ESTs, most were derived from the latex, and only a few from the bark and leaf. The recent transcriptome sequencing work from Xia et al. [24] and Triwitayakorn et al. [25] added millions of next-generation sequencing reads separately with both Illumina and Roche platforms. Other techniques such as microarray, serial analysis of gene expression (SAGE) and digital gene expression (DGE) have not been utilized so far in rubber tree. Molecular markers have been developed and employed for DNA fingerprinting in rubber tree [25-31], but the categories and numbers of molecular markers cannot keep up with the biological development of rubber tree.

During the last decade, a large number of genomic and transcriptomic sequences became available in model plants, such as *Arabidopsis* and rice, which has greatly improved our understanding of the growth and development in higher plants. For rubber tree, only limited genomic and transcriptomic sequences are available. The rubber tree genome is highly heterozygous because of its cross-pollination nature. Thus, transcriptome sequencing is an attractive alternative to whole-genome sequencing because transcriptome sequencing only focuses on the transcribed portions of the genome and avoids the non-coding and repetitive sequences that make up the majority of most eukaryotic genomes. The RNA-seq approach provides a cost-effective means for sequencing the transcriptome of an organism. Several transcriptome studies reported with RNA-seq techniques so far indicated that it was feasible for plant species to assemble and analyze the transcriptome with short-read sequence data [24,32-39]. Using the mixed materials of leaf and latex, Xia et al. [24] recently reported *de novo* transcriptome assembly of rubber tree with RNA-seq approach and submitted 37,432 unigenes.

Besides important roles in protecting plants, transporting water and nutrients and storing proteins, rubber tree bark contains the laticifers where latex is synthesized and stored. Compared with other tissues, bark is more important agricultural and biological organ in rubber tree. However, there are very limited data available for understanding the transcriptome of rubber tree bark. In this study, the transcriptome from rubber tree bark was sequenced with Illumina paired-end sequencing technology, the sequencing data were assembled and annotated, and EST-SSR markers were developed in rubber tree. To our knowledge, this is the first systematic report on the transcriptome of rubber tree bark. The research is essential and helpful to understand the transcriptome characterization of rubber tree bark. The transcriptome data generated from our study are very useful resources for gene annotation and discovery, molecular markers development, genomic and transcriptomic assembly, and microarrays development in rubber tree. In addition, the EST-SSR markers predicted and developed in this study will enrich the number of molecular markers, and facilitate genes mapping, linkage map development, genetic diversity analysis, and marker-assisted selection breeding in rubber tree.

## Results

### Illumina sequencing and *de novo* assembly

With the purpose of understanding the bark transcriptome of rubber tree, RNA was extracted from rubber tree barks and sequenced with Illumina paired-end sequencing technology. In this research, a total of 30,436,428 raw sequencing reads with the length of 100 bp were generated from a 200 bp insert library. Among them, 2,288,819 (7.52%) reads with significant homology to plant non-coding RNAs and 1,646,611 (5.41%) low-quality reads were firstly removed. The remaining more than 26 million high-quality reads were used to assemble the transcriptome of rubber tree bark with SOAPdenovo [40]. According to the overlapping information of high-quality reads, a total of 68,810 contigs were generated with an average length of 233 bp and a N50 of 291 bp, and the contigs with the length more than 500 bp accounted for about 8.56% (Table 1).

**Table 1:** Characteristics of assembled contigs, scaffolds and unigenes

**Nucleotides length (bp)**	**Contigs**	**Scaffolds**	**Unigenes**
75–500	62,918	30,245	15,576
501–1,000	4,821	5,253	5,143
1,001–1,500	831	1,509	1,504
1,501–2,000	205	449	448
2,001–2,500	26	73	71
2,501–3,000	6	7	8
>3,000	3	5	6
Total	68,810	37,541	22,756
N50 (bp)	291	464	592
Average length (bp)	223	364	485
Total nucleotides length (bp)	16,017,394	13,656,242	11,046,525

**Figure 1:**
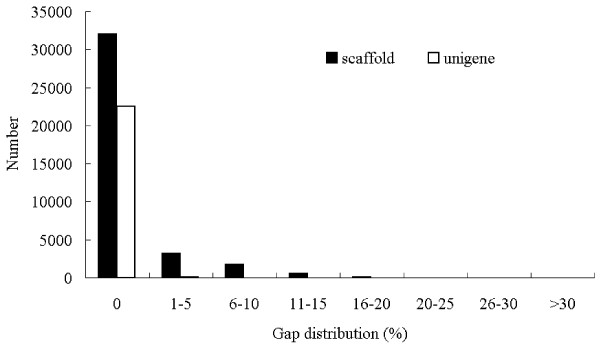
**Gap distribution of assembled scaffolds and unigenes.** The gap distribution (%) represents the percentage of the number of N divided by the sequence length of assembled scaffold or unigene.

With the help of paired-end reads, it is possible to identify the contigs derived from the same transcript and the distances among contigs within the same transcript. The reads were first mapped back to contigs, and then the contigs were assembled into scaffolds with “N” to represent unknown nucleotides between two contiguous contigs inferred from the paired-end information. As a result, 37,541 scaffolds were obtained with an average length of 364 bp and a N50 of 464 bp (Table 1). The scaffolds with the length over 500 bp accounted for about 19.43%. 32,038 scaffolds (approximately 85.34%) did not contain gap region, whereas the gap region lengths of 4,803 scaffolds (about 12.87%) were less than 10% of their corresponding scaffolds (Figure1).

To further shorten the remaining gaps, we gathered the paired-end reads with one end mapped on the unique contigs and the other end located in the gap region, and performed local assembly with the sequences on unmapped end to fill in the gaps within the scaffolds. In addition, Phrap was used to reduce the redundancy of scaffolds and extend the lengths of scaffolds. Such sequences without redundancy, containing the least amount of Ns and not being extended on either end, were defined as unigenes. With the steps mentioned above, 23,583 unigenes were finally obtained in this research. Of 23,583 unigenes, 827 unigenes indicated high identities to non-plant sequences and their BLAST results did not contain plant sequences, suggesting that those unigenes (about 3.51%) might represent contaminated sequences from other organisms (such as bacteria, fungi, etc.). After the removal of the contaminated sequences, 22,756 unigenes with a total length of about 11.05 Mb were obtained in this research. The assembled unigenes were submitted to the NCBI Transcriptome Shotgun Assembly (TSA) database, and assigned the accession numbers from JR344291 to JR366936. In addition, the assembled unigenes not conforming to the TSA standards (less than 200 bp in length, more than 10% N’s or containing greater than 14 N’s in a row) were shown in Additional file 1. The N50 and average length of unigenes were 592 and 485 bp, respectively (Table 1). The length of assembled unigenes ranged from 200 to 4,402 bp, and 7,180 unigenes (31.55%) had the length over 500 bp (Table 1). Among the assembled unigenes, 22,564 unigenes (about 99.16%) did not contain gap region, whereas only 192 unigenes (about 0.84%) were filled with Ns. The gap length distribution within the assembled unigenes was shown in Figure1. Xia et al. assembled the latex and leaf transcriptome with similar method used in this research [24], so the assembled unigenes from these two researches were compared with each other using local BLASTn program of BioEdit [41]. With E-value threshold of 1E-20, the number of unigenes specific to bark, latex and leaf were 5,162 and 27,027, respectively. Comparative analysis indicated that 21,741 unigenes from Xia et al. matched with 17,594 unigenes obtained from us, indicating that some unigenes obtained in our research have multiple hits against the unigenes reported by Xia et al. [24].

**Figure 2:**
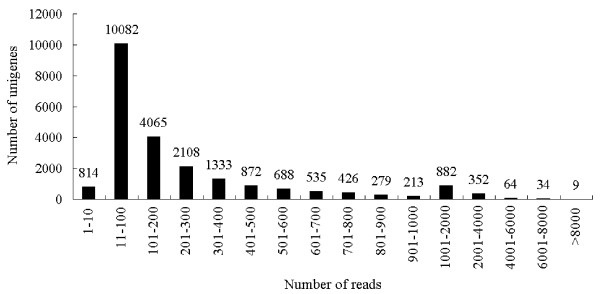
**Assessment of assembled quality.** The assembled quality of unigenes was assessed by the distribution of mapped reads within the assembled unigenes.

To evaluate the quality and coverage of the assembled unigenes, all the usable sequencing reads were realigned to the unigenes with a new alignment tool, SOAPaligner [42]. The sequencing depth of the assembled unigenes ranged from 0.19 to 75,645 folds, with an average of 46.33 folds in this research. As shown in Figure2, the read numbers mapped with per unigene (from 11 to 100) comprised the largest distribution, followed by 101–200 and 201–300. A total of 21,942 unigenes (about 96.42%) were realigned with more than 10 reads; 11,860 (approximately 52.12%) and 1,341 (about 5.89%) unigenes were remapped by more than 100 and 1,000 reads, respectively; whereas only nine unigenes were remapped with more than 8,000 reads (Figure2). These results suggested that the assembled unigenes were well overlapped by the sequencing reads.

### Functional annotation by searching against public databases

**Figure 3:**
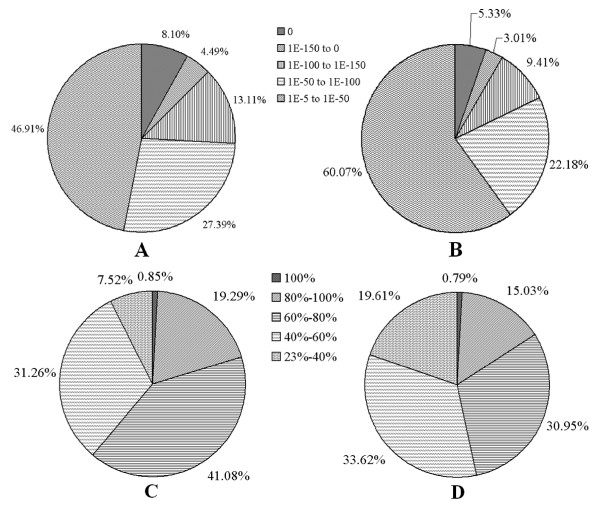
**Characterization of searching the assembled unigenes against Nr and Swissprot protein databases.** (**A**) E-value distribution of BLAST hits for the assembled unigenes with a cutoff of 1E-5 in Nr database. (**B**) E-value distribution of BLAST hits for the assembled unigenes with a cutoff of 1E-5 in Swissprot database. (**C**) Similarity distribution of the top BLAST hits for the assembled unigenes with a cutoff of 1E-5 in Nr database. (**D**) Similarity distribution of the top BLAST hits for the assembled unigenes with a cutoff of 1E-5 in Swissprot database.

For the validation and annotation of the assembled unigenes, all the assembled unigenes were searched against the NCBI non-redundant (nr) and Swissprot protein databases using BLASTx program with an E-value threshold of 1E-5. Among 22,756 unigenes, 16,520 (72.57%) were found to have significant similarity to 11,683 unique protein accessions in nr protein database. As expected, the less percentage was obtained when searching against Swissprot protein database. Of all the unigenes, 12,558 (55.19%) with significant identities to Swissprot proteins were matched with 7,539 unique protein accessions. Two studies have reported that the longer sequences were more likely to obtain BLAST matches in the protein databases [33,43], which was also validated by our results. About 89.77% of the unigenes over 500 bp in length had BLAST matches in the NCBI nr protein database, whereas only approximately 49.83% of unigenes shorter than 300 bp did in this research. The E-value distribution of the top hits revealed that 53.09% of the aligned sequences showed significant homology to entries in the nr database (<1E-50), and nearly 16.95% of the sequences showed more than 80% similarity (Figure3A and C). As for the BLAST results against the Swissprot database, the corresponding distributions of E-value (<1E-50) and similarity (>80%) were 39.93% and 15.82%, respectively (Figure3B and D). In total, BLAST searches identified 13,115 unique protein accessions from the nr and Swissprot protein databases, suggesting that this Illumina paried-end sequencing project generated a substantial fraction of rubber tree genes in this study.

Of all the 22,756 unigenes, 9 were remapped with more than 8,000 reads, which represented the most abundant transcripts in rubber tree bark (Table 2). Since rubber tree bark is regularly tapped for latex harvest, it is not surprising that some transcripts encoding the proteins associated with stress/defense response such as heat shock proteins and ascorbate peroxidase were highly expressed, which might play important roles in adapting to stress conditions [15,44,45]. Besides the stress/defense response proteins, two transcripts encoding s-adenosylmethionine synthetase and chalcone synthase were predominantly expressed. They were separately associated with s-adenosylmethionine metabolism and the biosynthesis of flavonoids, and performed a variety of functions in plants [46,47]. One transcript predominantly expressed probably encoded polyubiquitin, and recent studies reported that the polyubiquitin also fulfilled non-canonical functions besides protein degradation [48]. A highly expressed transcript, similar to checkpoint-like protein, was identified in this study. It is reported that CHK1 played vital role in DNA damage checkpoint [49]. In addition, the second abundant transcript with 20,460 mapped reads was similar to a hypothetical protein. It was noteworthy that the transcript mapped with 15,171 reads showed no significant similarity with the blastx and tblastx program, indicating that it might be novel sequence specific to rubber tree.

**Table 2:** Summary of most abundant unigenes in the transcriptome of rubber tree bark

**Unigene ID**	**No. of reads**	**Accession**	**E-value**	**Annotation**	**Source**
1725	174827	ABK29471.1	6e-19	CHK1 checkpoint-like protein	*H. armigera*
1124	20460	ACG27632.1	9e-16	hypothetical protein	*Z. mays*
1710*	15171	GR305569.1	3e-93	No	*H. brasiliensis*
22025	12755	AAP42157.1	2e-147	heat shock protein 70	*S. medusa*
22071	11715	AAA34124.1	0.0	polyubiquitin	*N. sylvestris*
18835	10115	AAQ08597.1	2e-72	heat shock protein	*H. brasiliensis*
48	9307	AAO14118.1	5E-136	ascorbate peroxidase	*H. brasiliensis*
20505	8197	XP_002512570.1	8E-123	s-adenosylmethionine synthetase	*R. communis*
20207	8095	ACN30003.1	3E-108	chalcone synthase	*V. rotundifolia*

* The information of accession, E-value, annotation and source was from the blastn program with NCBI EST database.

### Functional classification by GO and COG

**Figure 4:**
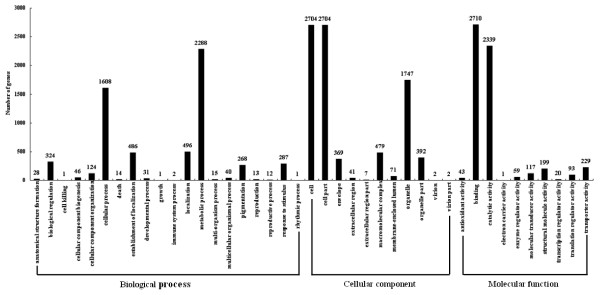
**Gene Ontology classifications of assembled unigenes.** 6,867 unigenes with significant similarity in nr protein databases were assigned to gene ontology classifications.

Gene Ontology (GO) is an international standardized gene functional classification system, and it is a useful tool to annotate and analyze the functions of a large number of genes and their products in any organism. Three ontologies, molecular function, cellular component and biological process, are provided in GO database. In this study, the Blast2GO program [50] was firstly used to analyze GO annotation of the assembled unigenes, and then the GO functional classifications of these unigenes were performed with WEGO software [51]. In total, 6,867 unigenes with BLAST matches to known proteins were assigned to GO classes with 20,413 functional terms. As shown in Figure4, the assignments to the cellular component made up the majority (8,561, 41.94%), followed by the biological process (6,085, 29.81%) and molecular function (5,767, 28.25%). Under the category of biological process, metabolic process (2,288, 37.60%) and cellular process (1,608, 26.43%) were prominently represented, indicating that some important metabolic activities and cell processes occurred in rubber tree bark (Figure4). Interestingly, 324 unigenes were assigned to the biological regulation. It was also noteworthy that 287 unigenes were involved in response to stimulus (Figure4). Under the classification of molecular function, binding (2,710, 46.99%) and catalytic activity (2,339, 40.56%) were separately the first and second largest categories, whereas other categories such as antioxidant activity, electron carrier activity, enzyme regulator activity, molecular transducer activity, etc. contained 718 unigenes only representing 12.45%. As for the cellular component, three categories, cell, cell part and organelle, were approximately 83.58% of cellular components; whereas only a few unigenes were assigned to extracellular region part, virion and virion part (Figure4).

**Figure 5:**
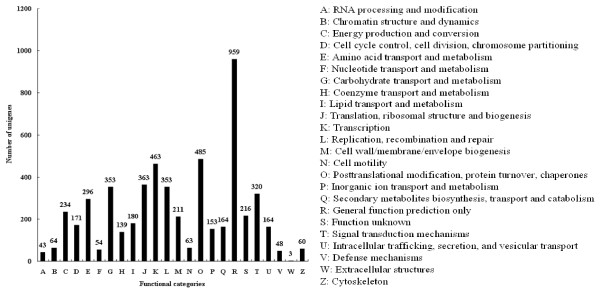
**Histogram presentation of COG classification.** All unigenes were aligned to COG database to predict and classify possible functions. Out of 16,520 unigenes with nr hits, 5,559 were assigned to 24 COG classifications.

The protein database of COGs is an attempt to phylogenetically classify the complete complement of proteins encoded in a complete genome. Each COG is a group of three or more proteins that are inferred to be orthologs, i.e., they are direct evolutionary counterparts. Therefore, the COG reflects one-to-many and many-to-many orthologous relationships as well as simple one-to-one relationships. Out of 16,520 unigenes with significant similarity to nr proteins in this study, 5,559 sequences were assigned to the COG classifications (Figure5). Among the 24 COG categories, the cluster for general function prediction only (959, 17.25%) was the largest group, followed by posttranslational modification, protein turnover and chaperones (485, 8.72%), transcription (463, 8.33%), translation, ribosomal structure and biogenesis (363, 6.53%), carbohydrate transport and metabolism (353, 6.35%), replication, recombination and repair (353, 6.35%), signal transduction mechanisms (320, 5.76%) and amino acid transport and metabolism (296, 5.32%), whereas the percentages of four groups, nucleotide transport and metabolism, defense mechanisms, RNA processing and modification and extracellular structures, were less than 1.00% (Figure5). Interestingly, 164 unigenes (about 2.95%) were classified into the group of secondary metabolites biosynthesis, transport and catabolism, suggesting that those important processes might occur in rubber tree bark.

### Functional classification by KEGG

The Kyoto Encyclopedia of Genes and Genomes (KEGG) Pathway is a collection of manually drawn pathway maps representing the knowledge on the molecular interaction and reaction networks. The pathway-based analysis is helpful to further understand the biological functions and genes interactions. To further analyze the transcriptome of rubber tree bark, all the unigenes were analyzed in KEGG pathway database. Out of the 22,756 unigenes, 12,097 ones (53.14%) with significant matches in the database were assigned to 5 main categories including 123 KEGG pathways ( Additional file 2). Among 5 main categories, metabolism was the biggest category (9,043, 74.75%), followed by genetic information processing (2,427, 20.06%), organismal systems (928, 7.67%), cellular processes (388, 3.21%) and environmental information processing (121, 1.00%). These results indicated that the active metabolic processes were underway in rubber tree bark. As shown in Additional file 2, the KEGG metabolism contained 12 categories, such as carbohydrate metabolism, nucleotide metabolism, the biosynthesis of other secondary metabolism, amino acid metabolism, lipid metabolism, energy metabolism, etc. As expected, all of the key genes involved in mevalonate (MVA) [11] and 2-C-methyl-D-erythritol 4-phosphate (MEP) pathways [12] were identified in metabolism pathway (Table 3).

**Table 3:** Unigenes of MVA and MEP pathways identified in this research

**Pathway**	**Gene name**	Accession number of matched genes*
MVA pathway	isopentenyl-diphosphate Delta-isomerase (IDI)	BAF98286.1 (2)
	acetyl-CoA C-acetyltransferase (AACT)	BAF98276.1 (2), ZP_08629444.1 (1), BAF98277.1 (1), AAL18924.1 (1)
	hydroxymethylglutaryl-CoA synthase (HMGS)	BAF98279.1 (1)
	hydroxymethylglutaryl-CoA reductase (HMGR)	P29057.1 (1), BAF98280.1 (1)
	mevalonate kinase (MVK)	AAL18925.1 (1),
	phosphomevalonate kinase (PMK)	BAF98284.1 (1), AAL18926.1 (1)
	diphosphomevelonate decarboxylase (MVD)	BAF98285.1 (2)
MEP pathway	1-deoxy-D-xylulose 5-phosphate synthase (DXS) 1-deoxy-D-xylulose 5-phosphate reductoisomerase (DXR)	XP_002533688.1 (2), ABD92702.1 (1), XP_002514364.1 (2), ZP_08629200.1 (1) ABQ53937.1 (1), AAS94121.1 (1), ABD92702.1 (1)
	2-C-methyl-D-erythritol 4-phosphate cytidylyltransferase (CMS)	BAF98292.1 (1)
	4-diphosphocytidyl-2 C-methyl-D-erythritol kinase (CMK)	BAF98293.1 (1)
	2-C-methyl-D-erythritol 2,4- cyclodiphosphate synthase (MCS)	BAF98295.1 (1)
	4-hydroxy-3-methylbut-2-en-1-yl diphosphate synthase (HDS)	BAF98296.1 (6)
	4-hydroxy-3-methylbut-2-enyl diphosphate reductase (HDR)	ACG55683.1 (1)

* Number of unigene with a hit in nr protein database.

In addition to the genes involved in the metabolic pathways, 2,531 unigenes were divided into the genetic information processing including transcription, translation, folding, sorting and degradation, replication and repair. A total of 928 unigenes were classified into organismal systems containing plant-pathogen interaction, plant circadian rhythm and natural killer cell mediated cytotoxicity, and the gene numbers of three sub-pathways were 761, 125 and 42, respectively. In addition, the categories of cellular process and environmental information processing separately included 388 and 121 unigenes ( Additional file 2). The functional classification of KEGG provided a valuable resource for investigating specific processes, functions and pathways involved in bark transcriptome of rubber tree.

### Development and characterization of EST-SSR markers

To further evaluate the assembly quality and develop new molecular markers, the 22,756 unigenes generated in this study were used to mine potential microsatellites that were defined as di- to nona-nucleotide motifs with a minimum of three repetitions. Using the Simple Sequence Repeat Identification Tool (SSRIT, http://www.gramene.org/db/markers/ssrtool), a total of 39,257 potential simple sequence repeat (SSR) were identified in 16,208 unigenes. Of the 16,208 unigenes, 6,549 and 9,659 unigenes contained one and more than one SSR, respectively (Table 4). The number of potential EST-SSR per unigene varied from 1 to 25 with an average of 2.42. Based on the SSR-containing sequences, 110 SSR sites were randomly selected to design EST-SSR primers with Primer Premier 6.0. The information of EST-SSR primers is shown in Additional file 3. Among the 110 primer pairs, 106 were successful in PCR amplification with genomic DNA from rubber tree, and the remaining four-pair primers failed to generate PCR products at various annealing temperatures and Mg^2+^ concentrations. Of the 106 working primer pairs, 73 PCR products showed specific amplification, among which 62 PCR products were as sizes as they expected, and the other eleven generated larger PCR products than expected, suggesting that the amplifying regions likely contained introns. The 33 PCR products indicated more than one band, which might result from the primer design or the high heterozygosity of rubber tree germplasm. The 106 primer pairs were further examined with 13 *Hevea* germplasm accessions as PCR templates. In total, 165 amplifying bands were detected by 106 primer pairs, and the number of amplifying bands per primer pairs ranged from one to seven, with an average of 1.56. With 13 *Hevea* germplasms as PCR templates, 61 and 45 primer pairs were found to be polymorphic and monomorphic, respectively. To check whether the EST-SSR markers developed in this study were novel, we searched the primer sequences of molecular markers previously reported in rubber tree against the target regions selected to design EST-SSR primers. The BLAST results indicated that 107 EST-SSR markers from our studies were firstly reported in rubber tree.

**Table 4:** Summary of EST-SSRs identified in rubber tree transcriptome

**Searching item**	**Numbers**
Total number of sequences examined	22,756
Total size of examined sequences (bp)	11,046,525
Total number of identified EST-SSRs	39,257
Number of EST-SSRs containing sequences	16,208
Number of sequences containing more than one EST-SSRs	9,659
Di-nucleotide	27,877
Tri-nucleotide	10,490
Tetra-nucleotide	430
Penta-nucleotide	203
Hexa-nucleotide	239
Hepta-nucleotide	13
Octa-nucleotide	2
Nona-nucleotide	3

**Figure 6:**
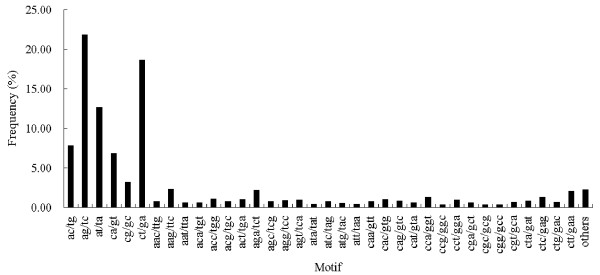
**Frequency distribution of EST-SSRs based on motif types.** Within the potential EST-SSRs, a total of 429 motif sequence types were identified. The frequency of main motif types was showed in this figure.

In addition, the characterizations of the potential 39,257 EST-SSRs were further analyzed in this study. The average length containing one SSR was about 281.39 bp. As shown in Table 4, the di-nucleotide repeats were the most abundant type (27,877, 71.01%), followed by tri- (10,490, 26.72%), tetra- (430, 1.10%), hexa- (239, 0.61%) and penta-nucleotide (203, 0.52%) repeats. Di- to nona-nucleotide motifs were further summarized for the number of repeat units. As shown in Table 5, the repeat unit of potential EST-SSRs mostly represented was 3, which accounting for 84.84% (33,304), followed by 4 (10.51%, 4,124), 5 (2.23%, 875) and 6 (0.84%, 330). A total of 98 potential EST-SSRs contained more than 14 repeat units, and all the motifs were di-nucleotide repeats (Table 5). Of 429 SSR motifs identified in this research, di-, tri-, tetra- and penta-nucleotide repeats were 6, 30, 99 and 88 types, respectively. As shown in Figure6, the AG/TC di-nucleotide repeat was the most abundant motif (8,569, 21.83%), followed by CT/GA (7,320, 18.65%), AT/TA (4,981, 12.69%), AC/TG (3,071, 7.82%), CA/GT (2,681, 6.83%) and CG/GC (1,255, 3.20%). The six types of nucleotide repeats mentioned above represented about 71.01%, whereas the remaining 423 types of nucleotide repeats only accounted for 28.99%.

**Table 5:** The distribution of EST-SSRs based on the number of repeat units

**No. of repeat unit**	**Di-**	**Tri-**	**Tetra-**	**Penta-**	**Hexa-**	**Hepta-**	**Octa-**	**Nona-**	**Total**
3	23,762	8,847	375	146	157	12	2	3	33,304
4	2,985	1,014	42	33	49	1	0	0	4,124
5	527	302	7	13	26	0	0	0	875
6	171	142	5	5	7	0	0	0	330
7	84	72	1	2	0	0	0	0	159
8	59	41	0	2	0	0	0	0	102
9	45	27	0	1	0	0	0	0	73
10	32	24	0	1	0	0	0	0	57
11	36	11	0	0	0	0	0	0	47
12	27	5	0	0	0	0	0	0	32
13	23	3	0	0	0	0	0	0	26
14	28	2	0	0	0	0	0	0	30
≥15	98	0	0	0	0	0	0	0	98

## Discussion

### Illumina paired end sequencing and assembly

Transcriptome sequencing is an effective method to obtain EST sequences that are essential for developing molecular markers and identifying novel genes. In the past decade, the development of various NGS technologies including Roche GS FLX and Solexa/Illumina platforms have made it possible to perform *de novo* transcriptome sequencing [52,53]. Among these sequencing methods, Roche GS FLX was widely utilized for *de novo* transcriptome sequencing in many organisms [25,43,54-63]. Compared with Roche GS FLX, the Illumina platform was mainly utilized in the organisms with reference genomes [64-67]. In recent years, an array of novel assembly methods have been developed and made short read assembly to be cost-effective. Therefore, *de novo* sequencing and assembly of transcriptome or genome have been successfully used for model [68-72] and non-model organisms [24,32-34,36-39,73-75]. Consistent with these reports, the results from this research also suggested that short reads from Illumina sequencing can be effectively assembled and used for gene identification and SSR marker development in non-model organisms. In this study, more than 26 million high-quality reads were used to assemble the transcriptome of rubber tree bark. This large dataset resulted in a relatively high sequencing depth, with an average of 46.33 folds. The assembly result indicated that the mean length of unigenes was 592 bp, which was longer than the results shown in previous studies [24,33,43,55,56,58]. By using similar method, Xia et al. (2011) assembled and analyzed the latex and leaf transcriptome of rubber tree. Compared with their studies [24], the mean and N50 sizes of contigs, scaffolds and unigenes generated in this research were longer with the exception of the N50 sizes of the unigenes. These results suggested that the transcriptome sequencing data from rubber tree bark were effectively assembled, which was further validated by the high proportion of unigenes matched with known proteins and the high PCR success rate of EST-SSR markers developed from the assembled unigenes.

Nevertheless, only about 38.63% reads were assembled into unigenes, which is lesser than the results reported by other research groups [33,43,55,58]. We propose that the high percentage of unassembled reads might have resulted from the following reasons such as relatively short reads generated by Illumina Genome analyzer, relative strict selection of assembly parameters (e.g., the K-mer size), low-abundant transcripts, simple repeat regions, alternative splicing, high heterozygous nature of rubber tree, etc. Although the high percentage of unassembled reads existed in this study, these unassembled reads were still an important resource for rubber tree research. To obtain better assembly results, other sequencing technologies (FLX-454, Sanger or other NGS technologies) should be utilized in combination with Illumina platform.

When all the usable sequencing reads were realigned to the assembled unigenes, an average sequencing depth of 46.33 folds was obtained in this research. However, of the 22,756 unigenes, more than 0.8% unigenes had a coverage depth of less than 1, which was partly due to the drawback to the de Bruijn graph approach used in SOAPdenovo program [76]. In de Bruijn approach, the reads were decomposed into k-mers, and then the sequence assembly was carried out. Therefore, the decomposing process likely causes the loss of information. In a few cases, only partial K-mers from the reads are utilized for the sequence assembly, which results in the assembled sequences not supported by the underlying reads. Moreover, the bubbles with high similarity are likely merged into one contig because they cannot be well distinguished due to the short read length and the lack of reference genome.

### Functional annotation of unigenes

For the transcriptome sequencing projects, the number of genes and the level of transcript coverage are usually important issues, but it is very difficult to estimate them due to the lack of a reference genome in this research. Using blast algorithm, we indirectly evaluated the transcriptome coverage breadth by estimating the number of unique genes. A large number of unigenes could be matched with unique known proteins in public databases, which implied that the Illumina sequencing project yielded a substantial fraction of unique genes from rubber tree. With the method reported by previous studies [33,43,57], if the number of genes in rubber tree was assumed to be commensurate with that of *Arabidopsis* (25,000), the annotated unigenes (13,115 unique protein accessions) would likely represent more than 52% of genes in rubber tree genome. The unigene number was less than that reported by Xia et al. [24] and Triwitayakorn et al. [25] separately with latex, leaf and shoot apical meristem as sequencing materials, but more than that reported by Chow et al. with latex as sequencing material [9]. In our research, the transcripts mostly expressed in bark were mainly associated with stress/defense response and secondary metabolism, whereas the most highly represented unique transcripts in latex and shoot apical meristem were involved in rubber biosynthesis, stress or defence responses and cyanogenic metabolism, respectively. These results indicated that the nature of abundant transcripts were distinct in different tissues, reflecting the unique transcriptomic signatures of different tissues.

In this study, a large number of unigenes were assigned to a wide range of GO categories and COG classifications (Figures 4 and 5), suggesting that the assembled unigenes represented a wide diversity of transcripts in rubber tree genome. Among three GO categories, cell and binding activity were the most abundant classes in cellular component and molecular function, respectively, which was consistent with the report from Xia et al. [24]. Triwitayakorn et al. also indicated that the majority category fell into binding activity among molecular function terms [25]. As for biological process, metabolic process was the largest group in our and Triwitayakorn’s studies [25], whereas cellular process in Xia’s work [24]. Among COG classifications, the second and third largest classifications unearthed in our work were separately posttranslational modification, protein turnover, chaperones and transcription, which was different from the report by Xia et al. [24]. In addition, the unigenes number in some COG classifications such as defense mechanisms, extracellular structures, RNA processing and modification, lipid transport and metabolism, translation, ribosomal structure and biogenesis, etc. were obviously different from the results of Xia et al. [24]. These results further confirmed that the bark transcriptome sequencing data unearthed new genes that were not identified by Xia et al., and vice versa. Therefore, assembling and analyzing the data from the transcriptome sequencing of various tissues would obtain more comprehensive and integrated set of transcriptome in rubber tree.

Among the KEGG pathways, the well represented pathways discovered in our study were spliceosome, plant-pathogen interaction, biosynthesis of plant hormones, biosynthesis of phenylpropanoids and ribosome, which was different from the results of Xia et al. [24]. Compared with the transcriptome of latex and leaves, there existed different inner-cell metabolic pathways in the transcriptome of rubber tree bark. Furthermore, lots of unigenes without hits in BLAST analyses likely corresponded to the untranslated regions, short sequences not containing a known domain, non-coding RNAs, or the potential rubber tree-specific genes. Generally speaking, such *de novo* transcriptome sequencing data can provide sufficient transcriptomic sequence information for identifying novel genes in rubber tree, which also confirm that high-throughput Illumina sequencing is an efficient, inexpensive and reliable tool for transcriptome characterization and gene discovery in non-model species.

### EST-SSR marker identification and characterization

It is well-known that EST-SSR marker is very important for the researches such as the assessment of genetic diversity, the development of genetic maps, comparative genomics, marker assisted selection breeding, etc. Only about several hundred EST-SSR markers have been developed until now [25,29,30], which limited the application of EST-SSR markers in rubber tree. The transcriptome sequencing provided plenty of sequences for developing numerous EST-SSR markers in rubber tree. In total, 39,257 potential EST-SSRs were identified in 16,208 unigenes. Although the selection criterions for developing EST-SSR markers in this study were different from the previous works, some identical results were obtained. If mono-nucleotide repeats were excluded, di-nucleotide repeats were the most abundant type, followed by tri- nucleotide repeats, which was consistent with previous reports [25,29,30]. The most abundant di- and tri-nucleotide motifs were AG/TC and AAG/TTC, respectively. These results were also coincident with previous reports except that the most abundant tri-nucleotide motifs was CTT/GAA in An’s research [25,29,30]. Of 110 pair primers randomly selected for PCR validation, 106 produced clear bands. The PCR success rate was higher than the results from Triwitayakorn et al. [25], An et al. [29] and Feng et al. [30] in rubber tree, but similar to Wang et al. [33] in sweet potato. Therefore, the 39,257 potential EST-SSRs identified in this research will provide a wealth of resource for developing EST-SSRs in rubber tree.

## Conclusion

In this work, we reported the transcriptome characterizations of rubber tree bark and provided valuable resources for new genes discovery and EST-SSR markers development, which will certainly accelerate the research progress in molecular biology of rubber tree. To our knowledge, this is the first attempt to assemble and characterize the transcriptome of rubber tree bark using Illumina paired-end sequencing method. Based on the transcriptome assembly, EST-SSRs were predicted and their characterizations were further analyzed. The 39,257 EST-SSRs predicted in this study laid a solid foundation for molecular marker development in rubber tree. These results fully demonstrate that Illumina paired-end sequencing is a fast and cost-effective approach for new genes discovery and molecular markers development in non-model organism, especially those with large genome.

## Methods

### Plant material, DNA and RNA extraction

The RY7-33-97, a high-yielding clone, was planted at the experimental farm of Chinese Academy of Tropical Agricultural Sciences in 1992. During the past 11 years, the plants were tapped once every 4 days for latex harvest, and 1.5% ethephon was applied to stimulate latex yield two days before tapping with once every three tappings. The bark samples were collected from healthy rubber trees, and then washed with diethyl pyrocarbonate treated water to remove the latex, and frozen in liquid nitrogen for RNA extraction. For Illumina sequencing, the total RNA was isolated from the bark tissues according to the method reported by Venkatachalam et al. [77]. RNA quality was detected with a 2100 Bioanalyzer (Agilent Technologies). The beads with oligo(dT) were used to isolate poly(A) mRNA from total RNA (Qiagen GmbH, Hilden, Germany).

To examine the polymorphism of EST-SSR markers, the leaves from thirteen *Hevea* germplasms, SCATC93-114, LCB1320, RRII118, PB86, RY-7-33-97, AC/F, RO/OP, MT/IT, MT/VB, *H. pauciflora* Muellet-Argoviensis, *H. spruceana* Mueller-Argoviensis, *H. benthamiana* Mueller-Argoviensis and *H. nitida* Mart var. *toxicodendroides*, were rinsed in deionized water and stored in −80°C freezer until DNA extraction. Two grams of leaves was ground in liquid nitrogen and genomic DNA was extracted using the CTAB method described by Doyle and Doyle [78]. DNA quantification was detected with a 2100 Bioanalyzer (Agilent Technologies) and gel electrophoresis analysis.

### cDNA library construction and sequencing

Illumina sequencing was performed at Beijing Genomics Institute (BGI)-Shenzhen, China according to the manufacturer’s instructions (Illumina, San Diego, CA). Firstly, mRNA with poly(A) tail was isolated from 20 μg total RNA using Sera-mag magnetic oligo (dT) beads (Illumina). To avoid priming bias, the purified mRNA was firstly fragmented into small pieces (100–400 bp) using divalent cations at 94°C for 5 minutes. With random hexamer primers (Illumina), the double-stranded cDNA was synthesized using the SuperScript double-stranded cDNA synthesis kit (Invitrogen, CA). The synthesized cDNA was subjected to end-repair and phosphorylation, and then the repaired cDNA fragments were 3′ adenylated with Klenow Exo- (3′ to 5′ exo minus, Illumina). Illumina paired-end adapters were ligated to the ends of these 3′-adenylated cDNA fragments. To select the proper templates for downstream enrichment, the products of ligation reaction were purified on 2% agarose gel. The cDNA fragments (about 200 bp) were excised from the gel. Fifteen rounds of PCR amplification were carried out to enrich the purified cDNA template using PCR primer PE 1.0 and 2.0 (Illumina) with phusion DNA polymerase. Finally, the cDNA library was constructed with 200 bp insertion fragment. After validating on an Agilent Technologies 2100 Bioanalyzer, the library was sequenced using Illumina HiSeq^TM^ 2000 (Illumina Inc., San Diego, CA, USA), and the workflow was as following: template hybridization, isothermal amplification, linearization, blocking, sequencing primer hybridization, and sequencing on the sequencer for read 1. After completion of the first read, the templates can be regenerated in situ to enable a second read from the opposite end of the fragments. Once the original templates are cleaved and removed, the reverse strands undergo sequencing-by-synthesis.

### Data filtering and *de novo* assembly

Before the transcriptome assembly, we carried out a stringent filtering process of raw sequencing reads. The reads with more than 10% of bases with a quality score of Q < 20, non-coding RNA (such as rRNA, tRNA and miRNA), ambiguous sequences represented as “N” and adaptor contamination were removed; moreover, we also discarded the reads that do not pass the Illumina failed-chastity filter according to the relation “failed-chastity ≤ 1”, with a chastity threshold of 0.6 on the first 25 cycles.

*De novo* transcriptome assembly was performed by de Bruijn graph and SOAPdenovo with the default settings except K-mer value [40]. The high-quality reads were loaded into the computer, and then de Bruijn graph data structure was used to represent the overlap among the reads. In this step, we firstly broke down all reads into 29 mers which were used as nodes to construct the de Bruijn graph and two reads overlapping 28 bp were connected. To reduce the graph complexity, the tiny repetitive sequences shorter than the read length in the graph were resolved by read paths; the short tips with the lengths less than 2 *K* (58 bp) and lower frequency than other alternative paths were clipped in the graph; the low-coverage links that appeared only once along with their related edges were also filtered; the detected bubbles were merged into a single path if the sequences of the parallel paths were a single base pair difference or had fewer than four base pairs difference with >90% identity. After these steps, the connections on simplified graph were broke at repeat boundaries, and then the unambiguous fragments produced from the graph were defined as contigs.

After obtaining the contig sequences, we realigned the short reads onto the contigs and utilized the paired-end information to estimate the order and interval distance of two contigs. The repeat contigs with multiple and conflicting connections to the unique contig were masked, and the remaining contigs with compatible connections were assembled with Ns representing unknown nucleotides according to the pair-end linkage and insert size information. Following the above processes, the contigs with compatible connections to each other were constructed into scaffolds. Finally, paired-end information was used to retrieve the read pairs with one read well aligned on the contig and another read located in the gap region, and then a local assembly for the collected reads was carried out to make the scaffolds with least Ns. The scaffolds were further assembled to reduce the redundancy of scaffolds and extend the lengths of scaffolds using Phrap (http://bozeman.mbt.washington.edu/phrap.docs/phrap.html). After the steps mentioned above, the sequences obtained without redundancy, containing the least amount of Ns, and not being extended on either end were defined as unigenes. To evaluate the depth of coverage, all usable reads were realigned to the unigenes using SOAPaligner with the default settings [42].

### Gene annotation and analysis

All unigenes were utilized for homology searches against various protein databases such as NCBI nr (http://www.ncbi.nlm.nih.gov/), Swissprot (http://www.expasy.ch/sprot/), the COG (http://www.ncbi.nlm.nih.gov/cog/), and the KEGG pathway (http://www.genome.jp/kegg/) with BLAST program (E-value < 1E-5), and the best aligning results were selected to annotate the unigenes. If the aligning results from different databases are in conflict with each other, the results from nr database were preferentially selected, followed by Swissprot, KEGG and COG database.

To further annotate the unigenes in this research, the Blast2GO program [50] was used to get GO annotation according to molecular function, biological process and cellular component ontologies (http://www.geneontology.org/). The unigene sequences were also aligned to the COG database to predict and classify possible functions. Pathway assignments were performed according to KEGG pathway database [79].

### Development and detection of EST-SSR markers

The SSRIT was used to identify microsatellites in the unigenes. In this research, EST-SSRs were considered to contain motifs with two to nine nucleotides in size and a minimum of 3 contiguous repeat units. The frequency of EST-SSR refers to the cDNA sequences length containing one SSR. The primer premier 6.0 was used to design PCR primers. In total, 110 pairs of primers were designed ( Additional file 3) and validated by PCR reactions. The PCR amplification was carried out as follows: PCR mixtures were 94°C for 4 min, followed by 35–40 cycles of 94°C for 30 s, 55-60°C for 30 s and 72°C for 2 min. The final extension was performed at 72°C for 10 min. The PCR products were analyzed by electrophoresis on 8.0% non-denaturing polyacrylamide gels and silver stained [80]. The band sizes were determined against DNA ladder.

## Competing interests

The authors declare that they have no competing interests.

## Authors’ contributions

All the authors read and approved the final manuscript. DL supervised the experiments, and conducted sequence analysis, functional annotation, SSR prediction and primer design. In addition, he drafted and revised the manuscript. ZD and BQ participated in data analyses and SSR validation. XL conducted samples collection and preparation. ZM participated in data analyses and revised the manuscript.

## Supplementary Material

Additional file 1 The nucleotide sequences of assembled unigenes without the TSA standards. Click here for file

Additional file 2 **Summary of the unigenes annotated to the reference canonical pathways in the KEGG database.** The file contains KEGG pathway and the number of unigenes involved in the KEGG pathway. Click here for file

Additional file 3 **The characterizations of EST-SSR markers.** The file contains unigenes ID, primer sequences, product size, motif and repeat number, SSR length, polymorphism and amplification result. Click here for file
